# Pancreatic ductal adenocarcinoma in a patient with pancreas divisum and gastrointestinal duplication cyst: a case report

**DOI:** 10.1186/s40792-021-01279-4

**Published:** 2021-08-25

**Authors:** Naoto Takahashi, Hiroyuki Nitta, Akira Umemura, Hirokatsu Katagiri, Shoji Kanno, Daiki Takeda, Kenji Makabe, Satoshi Amano, Masao Nishiya, Noriyuki Uesugi, Tamotsu Sugai, Akira Sasaki

**Affiliations:** 1 Department of Surgery, Iwate Medical University, 2-1-1 Idaidori, Yahaba, 028-3695 Japan; 2 Department of Molecular Diagnostic Pathology, Iwate Medical University, 2-1-1 Idaidori, Yahaba, 028-3695 Japan

**Keywords:** Pancreas divisum, Pancreatic ductal adenocarcinoma, Gastric duplication cyst, Distal pancreatectomy, Gemcitabine, Nab-paclitaxel, S-1

## Abstract

**Background:**

The complication of duplication of alimentary tracts and pancreas divisum (PD) is a rare malformation and the development of pancreatic ductal adenocarcinoma (PDAC) in this malformation is also extremely rare. There have been some reports of complication of malignancy in a gastric duplication cyst (GDC) and PD. However, there have been no reports of complication of PDAC in cases with GDC and PD.

**Case presentation:**

A 54-year-old woman was followed up at the previous hospital due to a history of ovarian endometrial adenocarcinoma. She also had a surgical history of partial excision for a GDC and pancreatic tail of PD in her childhood. A gynecological follow-up computed tomography (CT) examination revealed the pancreatic body tumor and the bifurcated main pancreatic duct dilatation. Furthermore, magnetic resonance cholangiopancreatography also revealed that the ventral main pancreatic duct communicated with the GDC. The initial levels of tumor markers were high, but we could not achieve preoperative histopathological diagnosis. The preoperative diagnosis was PDAC occurring in a case with PD and GDC. She received two courses of neoadjuvant chemotherapy with gemcitabine and nab-paclitaxel. A CT examination after neoadjuvant chemotherapy revealed the shrinkage of the tumor, and then we performed distal pancreatectomy with splenectomy and GDC resection. A histopathological examination revealed invasive PDAC and lymph node metastases; pathological staging was T1N1M0, stage III. Furthermore, PD and GDC were also histopathologically detected. The postoperative course was uneventful, and she was discharged on the postoperative day 25. She received S-1 monotherapy for 6 months, and no recurrence has been detected at 1 year after radical resection.

**Conclusions:**

We herein presented an extremely rare combined case of PD, GDC and PDAC. We successfully treated it by neoadjuvant chemotherapy and distal pancreatectomy with GDC resection, and postoperative chemotherapy.

## Background

Duplication of the alimentary tracts is a rare developmental anomaly that may occur anywhere between the mouth and rectum, with 4–9% occurring in the stomach [1]. Duplication of the stomach sometimes forms a gastric duplication cyst (GDC) and complicates malignancies [2]. Pancreas divisum (PD), which is a common congenital anomaly of the pancreas, is the result of non-fusion of the ventral and dorsal pancreatic duct systems [3]. Common symptoms of PD are abdominal pain, vomiting and pancreatitis. Patients with PD also have a greater risk of developing a pancreatic tumor [3].

The combination of PD and GDC is a rare condition. Furthermore, complication of pancreatic ductal adenocarcinoma (PDAC) is an extremely rare condition. We herein report an extremely rare case of PDAC that occurred in a patient with PD and GDC.

## Case presentation

**Fig. 1:**
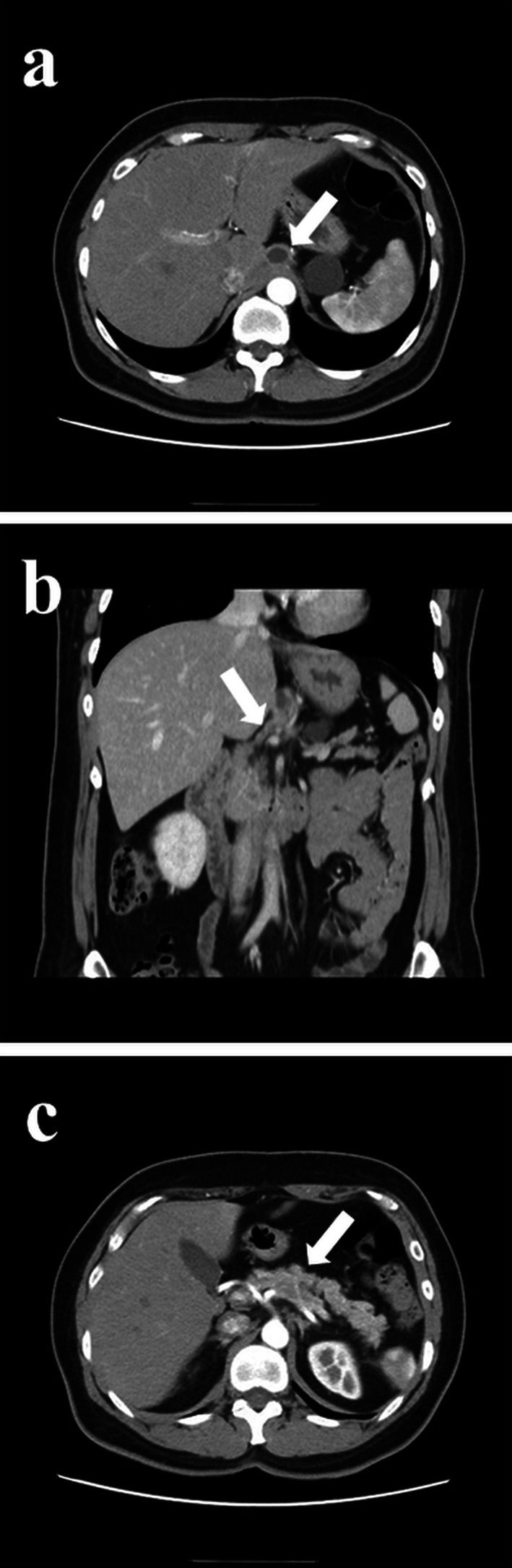
CT images before neoadjuvant chemotherapy. **a** A cystic nodular lesion observed beside the crus (white arrow), and cystic formation of PD was observed. **b** The nodular lesion was the GDC and connected to the pancreas (white arrow). **c** A 16-mm mass with poor contrast effect on the pancreatic body was detected (white arrow)

**Fig. 2:**
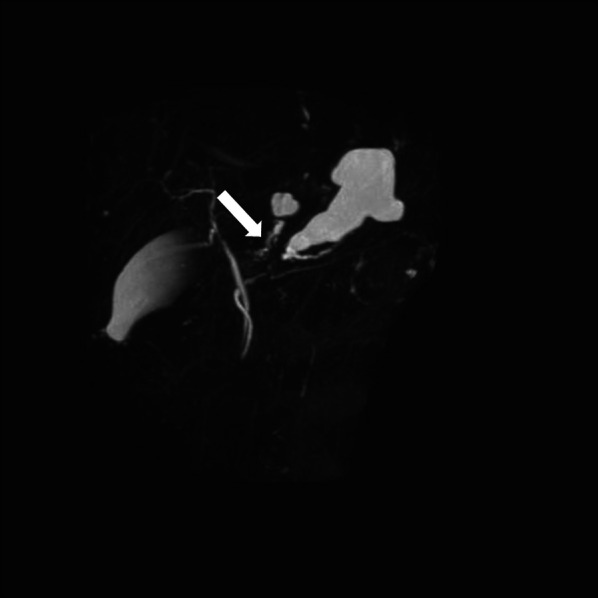
MRCP image before neoadjuvant chemotherapy. A cystic lesion protruding outside the ventral pancreas, continuous with the bifurcated main pancreatic duct. The nodular lesion was connected to the pancreatic duct (white arrow)

**Fig. 3:**
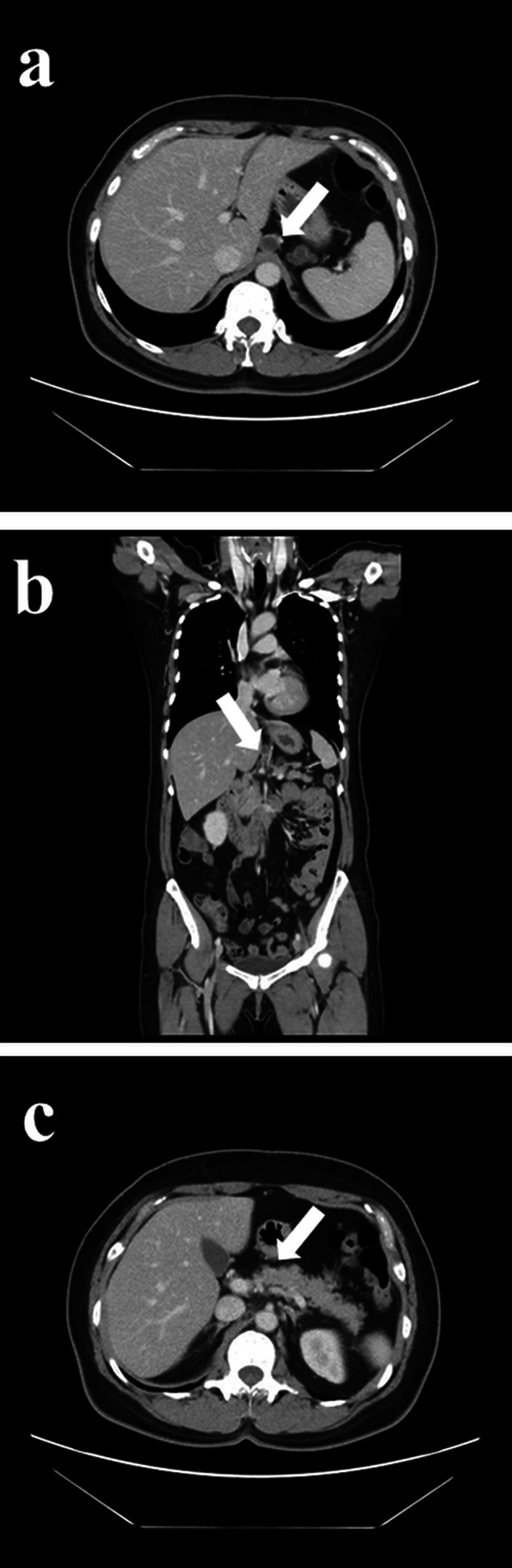
CT images after neoadjuvant chemotherapy. **a** The shrinkage of both GDC and cystic formation of the pancreatic duct was observed (white arrow). **b** The fistula between the GDC and the pancreas could be observed (white arrow). **c** The tumor became unclear after neoadjuvant chemotherapy (white arrow)

**Fig. 4:**
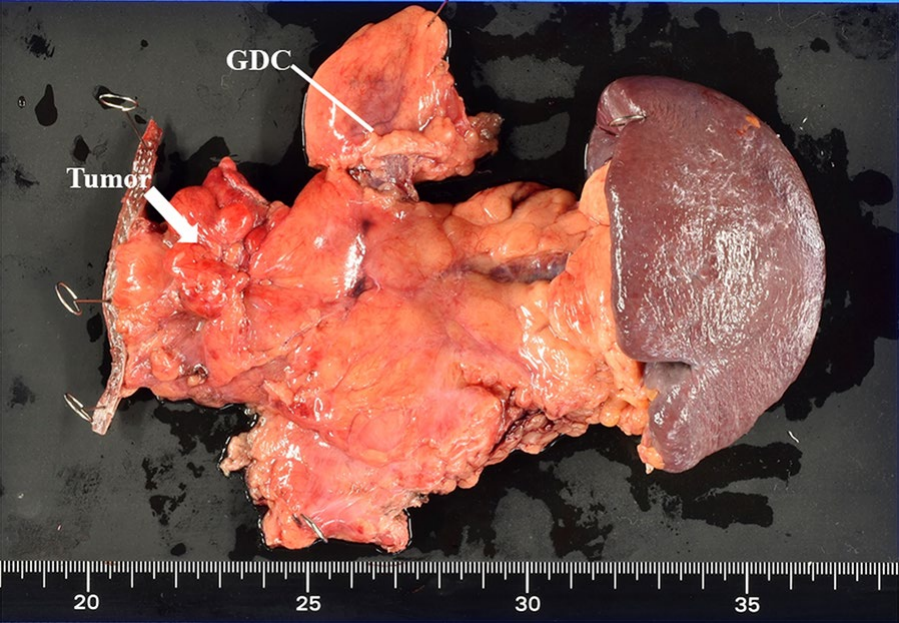
Macroscopic findings of resected specimens. The tumor was located at the pancreas body and distortion of the pancreas capsule was also observed (white arrow). The GDC was resected en bloc

**Fig. 5:**
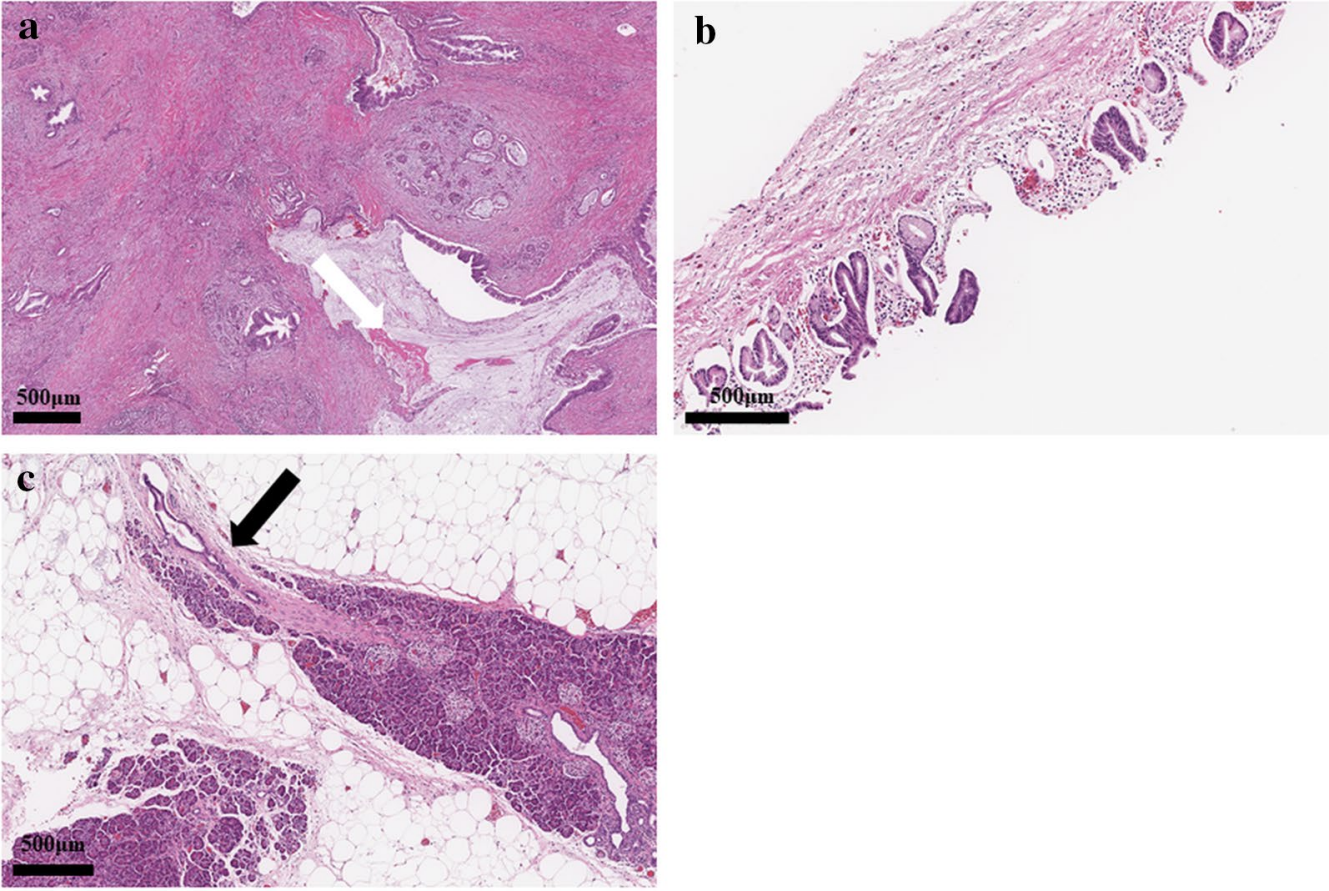
Microscopic findings of resected specimens. **a** The tumor was composed of moderately differentiated invasive PDAC, and carcinoma in situ and high-grade pancreatic intraepithelial neoplasia surrounded the tumor (white arrow). **b** Glandular fossa epithelium and gastric fundic gland were observed on the luminal side of the GDC, and mucosal muscle plate and muscle layer were also observed. **c** The fistula between the GDC and pancreas was histopathologically observed (black arrow)

**Fig. 6:**
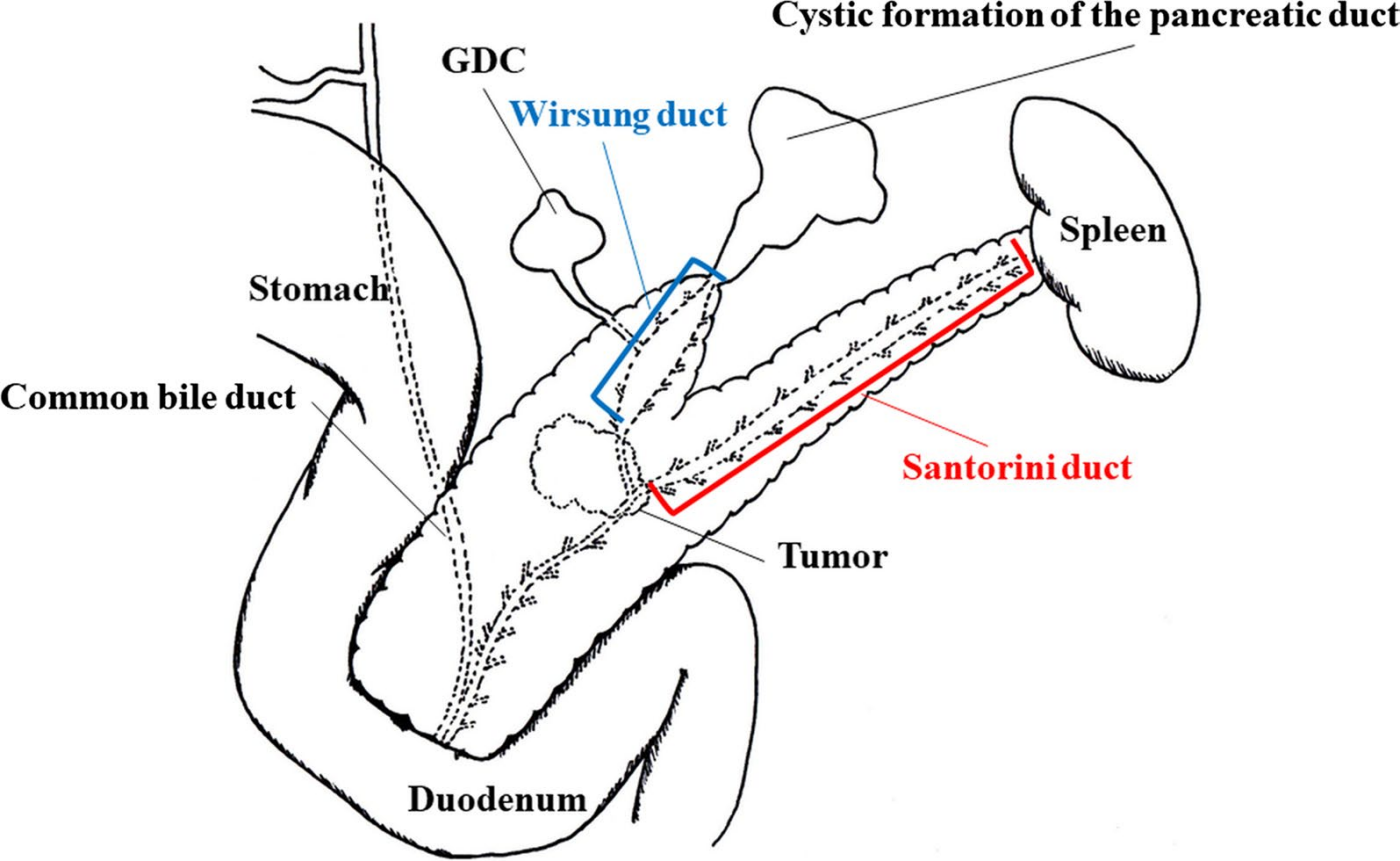
The schema of anatomical features in this case. In this case, PDAC occurred at the junction of pancreatic ducts of PD, and the disruption by PDAC induced the GDC dilatation and cystic formation of the pancreatic duct

A 54-year-old woman was followed up by the gynecological department of the previous hospital due to a history of left ovarian endometrial adenocarcinoma. She also had a history of partial excision for GDC and PD in her childhood, but there was no detail of the surgical record. A follow-up computed tomography (CT) examination revealed a cystic nodular lesion beside the crus (Fig. 1a). At first, we believed this nodule to be a metastatic lymph node due to some malignant tumors. However, we could retrospectively recognize that it was the GDC and the GDC was connected to the pancreas (Fig. 1b). In addition, PD, dilatation of the main pancreatic duct and cystic formation (Fig. 1a), and a 16-mm mass with poor contrast effect on the pancreatic body were detected (Fig. 1c); therefore, PDAC with lymph node metastasis was suspected. Laboratory findings also showed rising carbohydrate antigen 19-9 (CA19-9; 207.0 U/mL), Duke pancreatic monoclonal antibody type 2 (DUPAN-2; 230.0 U/mL) and s-pancreas-1 antigen (SPan-1; 65.0 U/mL) except for carcinoembryonic antigen (2.0 ng/mL). Magnetic resonance cholangiopancreatography (MRCP) showed a cystic lesion protruding outside the ventral pancreas, continuous with the bifurcated main pancreatic duct (Fig. 2). Observing that the nodular lesion was connected to the pancreatic duct, we recognized that the nodular lesion beside the crus was not a metastatic lymph node, but a GDC (Fig. 2). We considered performing endoscopic ultrasound-guided fine needle aspiration to clarify the histopathological diagnosis. However, we abandoned it due to the possibility of rupture of the pancreatic cystic formation and dissemination of tumor cells. On the other hand, pancreatography and pancreatic juice cytology via papilla of Vater were also avoided because cannulation and pancreatography might increase the intraductal pressure. From these examinations, she was diagnosed with PDAC in pancreas body, T1N0M0 Stage IA. As neoadjuvant chemotherapy for PDAC, she received two courses of nab-paclitaxel and gemcitabine therapy. A CT examination after neoadjuvant chemotherapy revealed that the GDC shrank due to drainage of the content (Fig. 3a) and the fistula between the GDC and the pancreas could be observed (Fig. 3b). Shrinkage of the tumor and mitigation of cystic formation of the main pancreatic duct were also observed (Fig. 3a, c). In addition, CA19-9, DUPAN-2, and Span-1 decreased to within normal limits; therefore, we planned to perform a distal pancreatectomy as a radical resection for PDAC with a concomitant resection of the PD and GDC. We performed the distal pancreatectomy with D2 lymphadenectomy and extracted the PD and GDC. Massive adhesion due to repeated laparotomy was observed; thus, we had to perform adhesiotomy before resection. It was considered that the severe inflammation and adhesion between the stomach and the pancreas were induced by the pancreatic cystic formation, but we could not find the cystic lesion during the operation. We employed a linear stapler for pancreas parenchymal transection. The operating time and blood loss were 237 min and 188 mL, respectively. Macroscopic findings of the resected specimen revealed that a hard tumor 15 × 11 mm in size was located at the pancreas body (Fig. 4). Histopathological examination revealed that the tumor was composed of moderately differentiated invasive PDAC, and carcinoma in situ and high-grade pancreatic intraepithelial neoplasia surrounded the tumor (Fig. 5a). Tumor cells were almost viable; therefore, the therapeutic effect of neoadjuvant chemotherapy was evaluated as grade 1b. There were on-section lymph node metastases and lymphatic ductal invasion. The histopathological diagnosis was T1N1M0, stage IIB. With regard to GDC, glandular fossa epithelium and gastric fundic gland were observed on the luminal side of the cyst, and mucosal muscle plate and muscle layer were observed in the deep part of the cyst (Fig. 5b). The fistula between the GDC and pancreas was observed (Fig. 5c). However, histopathological examination could not find the pancreatic cystic lesion that had shrunk after neoadjuvant chemotherapy. The anatomical features of this case are summarized in Fig. 6. The patient’s postoperative course was uneventful, and she was discharged on postoperative day 25. We performed eight courses of adjuvant S-1 monotherapy and she has been followed up without recurrence for 1 year.

## Discussion

GDCs are a congenital malformation that can occur in the entire gastrointestinal tract, with a reported incidence of 1 in 4500 to 10,000 births [4]. In general, GDCs are almost twice as common in women, and 20% have coexistence of other intestinal duplications. GDCs have been reported to be caused by developmental abnormalities of the foregut, and the two most widely supported hypotheses are Bremer’s recanalization inhibition and McLetchie’s chordal separation [5]. Communication or fistulation between the GDC and true gastric lumen is very rare, and poorly differentiated epithelium in the GDC wall may lead to carcinogenesis [2]. Therefore, surgical resection is recommended for patients with GDC. In this patient, the remnant GDC observed by CT examination was initially thought to be a metastatic lymph node due to PDAC. However, MRCP revealed the remnant to be GDC. From our experience, when a capsulated low-density tumor is observed around the stomach during work-up for other diseases, GDC should be taken as the differential diagnosis.

On the other hand, PD is also a rare anatomical abnormality, and its frequency has been reported to be 6–10% in patients with a history of endoscopic retrograde cholangiopancreatography and in autopsy cases [6]. Pancreatic malformations can be classified into migration and fusion anomalies. Fusion anomalies include those in which the dorsal pancreatic bud (including the Santorini duct) and the ventral pancreatic bud (including the Wirsung duct) do not fuse properly, causing the pancreas to divide. When the primary channels of multiple pancreatic ducts are formed, PD can result [6]. In this case, preoperative CT and MRCP showed a bifurcation of the main pancreatic duct, a pancreatic tail dividing into two parts, and a connection from the ventral side of the main pancreatic duct to the GDC. The histopathological results of the resected specimen showed that the GDC was connected to the pancreatic duct of the PD. In this case, duplicated pancreas tails and peripheral pancreatic ducts were observed, however, the embryological mechanism of duplicated pancreatic tails with peripheral pancreatic ducts has not been clarified yet. The usual concept of PD is determined by the incomplete fusion of the Santorini duct and the Wirsung duct at the duodenal side; therefore, duplication of pancreatic ducts and patency of the minor duodenal papilla are typical features. The ventral pancreatic bud usually rotates and fuses at the caudal side of the dorsal pancreatic bud. However, it seemed that the ventral pancreatic bud fused to the cranial side of the dorsal pancreatic bud in this case. We hypothesized that the ventral pancreatic bud might overhang toward the cranial side during the rotation because the ventral pancreatic bud was drawn by the GDC. As a result, the Wirsung duct properly fused with the Santorini duct at the duodenal side. However, the peripheral side of the ventral pancreatic bud did not fuse; therefore, bifurcated pancreatic ducts and duplicated pancreatic tails were formed. From these embryological backgrounds, this situation is defined as inverted PD [7, 8].

In addition, PDAC appeared at the junction of the PD and the fistula of the GDC.

Duplicated stomach has been found to be continuous with the main pancreatic duct in 78% of cases [9]. Traverso et al. reported that recurrent pancreatitis may occur due to secretion inflow when the duplicated stomach is continuous with the main pancreatic duct [10]. There are some reports of cystic carcinogenesis in pancreatic-associated GDCs, however, there has been only one reported case of PDAC with pancreatic-associated GDC, reported by Chiu et al. [11]. Furthermore, there have been no reports of cases involving PD. PD itself may have the potential to induce hepatobiliary malignancy or intraductal papillary mucinous neoplasms due to continuous stimulation by the pancreatitis [12, 13]. It is suggested that recurrent pancreatitis in pancreatic-associated GDCs may be related to mucus inflow or hemorrhage from the cyst to the communicating pancreatic duct [14]. Due to the risk of malignant degeneration of the cyst and pancreas, it is necessary to consider total resection of the pancreas and cysts rather than cyst drainage. As for this case, there were no risk factors for PDAC, such as diabetes mellitus or a history of abdominal pain due to pancreatitis or alcohol abuse. However, the patient may have had subclinical recurrent chronic pancreatitis due to increased risk of pancreatitis caused by PD and secretion inflow from the GDC. It is possible that the recurrent chronic pancreatitis may have triggered the development of PDAC at the junction of the pancreatic duct.

## Conclusion

We experienced an extremely rare case of PDAC with PD and GDC. Detailed image retrieval is required for the diagnosis of PD and GDCs, and if the patient is diagnosed with PD, surgical resection may be considered due to the possibility of malignant changes. In this case, asymptomatic chronic pancreatitis due to pancreatic-associated GDC triggered the carcinogenesis of PDAC, and PDAC occurred at the junction of the PD and the fistula to the GDC.

## Data Availability

Not applicable.

## Abbreviations

GDC: Gastric duplication cyst
PD: Pancreas divisum
PDAC: Pancreatic ductal adenocarcinoma
CT: Computed tomography
CA19-9: Carbohydrate antigen 19-9
DUPAN-2: Duke pancreatic monoclonal antibody type 2
Span-1: S-pancreas-1 antigen
MRCP: Magnetic resonance cholangiography
